# In Silico Non-Homologous End Joining Following Ion Induced DNA Double Strand Breaks Predicts That Repair Fidelity Depends on Break Density

**DOI:** 10.1038/s41598-018-21111-8

**Published:** 2018-02-08

**Authors:** N. T. Henthorn, J. W. Warmenhoven, M. Sotiropoulos, R. I. Mackay, N. F. Kirkby, K. J. Kirkby, M. J. Merchant

**Affiliations:** 1 Division of Molecular and Clinical Cancer Sciences, Faculty of Biology, Medicine and Health, University of Manchester, Manchester, UK; 2 Christie Medical Physics and Engineering, The Christie NHS Foundation Trust, Manchester, UK; 3 The Christie NHS Foundation Trust, Manchester, UK

## Abstract

This work uses Monte Carlo simulations to investigate the dependence of residual and misrepaired double strand breaks (DSBs) at 24 hours on the initial damage pattern created during ion therapy. We present results from a nanometric DNA damage simulation coupled to a mechanistic model of Non-Homologous End Joining, capable of predicting the position, complexity, and repair of DSBs. The initial damage pattern is scored by calculating the average number of DSBs within 70 nm from every DSB. We show that this local DSB density, referred to as the cluster density, can linearly predict misrepair regardless of ion species. The models predict that the fraction of residual DSBs is constant, with 7.3% of DSBs left unrepaired following 24 hours of repair. Through simulation over a range of doses and linear energy transfer (LET) we derive simple correlations capable of predicting residual and misrepaired DSBs. These equations are applicable to ion therapy treatment planning where both dose and LET are scored. This is demonstrated by applying the correlations to an example of a clinical proton spread out Bragg peak. Here we see a considerable biological effect past the distal edge, dominated by residual DSBs.

## Introduction

The induction of DNA double strand breaks (DSBs) is the mechanism by which radiation kills cells; with DSBs either repaired, misrepaired, or left unrepaired (residual). Misrepaired DSBs, considered potentially lethal lesions^1^, can lead to chromosome-type or chromatid-type aberrations, which are experimentally measurable^2^. These aberrations cause genomic instability, contributing to the chance of cell death^3^ and development of secondary cancers. Residual DSBs are likely to cause cell cycle arrest if detected by the various cell cycle checkpoints. The unresolved residual DSBs will cause cell death, and residuals that are missed by the G2/M checkpoint will cause the cell to undergo mitotic catastrophe. For this reason residual DSBs are conventionally considered to contribute substantially to the likelihood of cell death^3^.

A difference in the cell killing effectiveness between radiation qualities has previously been noted in the literature^4,5^, where a greater effect is seen for higher linear energy transfer (LET) radiations. Clinically it is important to understand this difference as it impacts the required dose for tumour control between radiation qualities, such as photons and protons. In practice, this is accounted for by use of the proton relative biological effectiveness (RBE), which is the ratio of proton dose relative to photon dose to achieve iso-effectiveness. An RBE value of 1.1 is in clinical use to give the physical proton dose that is biologically equivalent to the required photon dose^6,7^. However, it has been shown that the proton RBE is variable depending on a number of factors including dose, fraction, α/β ratio, and LET^8^. Marshall *et al*.^9^ have recently shown that consideration of RBE variation along a proton Spread Out Bragg Peak (SOBP) results in increases in both the delivered dose and range when compared to a constant RBE. To fully exploit the beneficial dose deposition characteristics of protons over photons, the variable RBE must be understood and incorporated into Treatment Planning Systems (TPS).

RBE has been linked to LET, where the LET of a particle is defined as the energy deposited per unit length. An increase in LET is equivalent to an increase in the number of ionisations in the same distance. In the context of particle therapy, a higher LET causes a greater number of DSBs^10^ as well as a higher complexity of DSB^11^. The proximity between DSBs is also dependent on the LET, with high LET leading to more proximal breaks due to the closer spacing between energy depositions^12^. Assuming irradiation in the same cell line and cell cycle phase, i.e. same α/β or simplistically “the same biology”, then the differences in the success or failure of the cell’s DNA damage response (DDR) depends only on these three DSB factors; the number of, complexity of, and proximity of DSBs, regardless of the radiation quality used. Similar statements have been expressed in other work, for example the Local Effects Model (LEM)^13,14^.

Break complexity and proximity have both been suggested to influence misrepair^15^. It has been proposed that either a class of DSB exists that has a propensity for causing aberrations^16,17^, or that misrepair occurs through pairwise interaction of DSBs dependent on their proximity^18^. The influence of DSB proximity on chromosome aberrations has been reviewed previously^19^ and has been investigated experimentally^20^. Determining the distance over which these DSB interactions occur is of importance for mechanistic models of DNA repair^21,22^. However, predictions for the value of this DSB separation varies within the literature; with values ranging from 0.25 µm to 1.30 µm^12,23–27^. The motion of DSB ends has a large impact on this value and is a debated topic in the literature. Some experimental work has shown that DSB ends in metazoan have limited motion in the nucleus and are unable to explore the entire volume^28,29^, travelling less than 0.5 µm in a 10 µm diameter nucleus^30^. On the other hand, evidence has also been published to suggest greater mobility of DSB ends^31^, particularly along the tracks of damage caused by heavy ions^31,32^. More directed motion has also been suggested by Neumaier *et al*., reporting the observation of repair centres in human cells^33^. To fully understand the consequence of how DSB end mobility leads to chromosome aberrations a more detailed repair model must be used.

Averbeck *et al*.^34^ have shown that DSBs induced across the carbon Spread Out Bragg Peak (SOBP) are efficiently repaired, although there is an increase in cell death. The authors attribute this increase to a greater number of misrepaired DSBs, highlighting the role of misrepair in RBE. It is known that, for the same cell line, the RBE with different ion species at the same LET (iso-LET) is substantially different^35^. This implies that the change in RBE of ions arises from differences in track structure^36^, the energy deposition pattern at the nanoscale. This can explain both DSB complexity and proximity, information that is not fully encompassed by the LET alone. The concept of track structure leading to biological outcomes is a core principle of the emerging field of nanodosimetry^37^. To our knowledge, no study has assessed the difference in misrepair induction between ion species and across a range of LET relevant to the clinical setting.

In this work, Monte Carlo simulations are used to predict the damage and repair of cells irradiated by protons, alpha particles, and carbon ions at a range of iso-LET. By scoring the average number of DSBs within close proximity of each other we are able to show a linear relation to misrepair across all the radiation qualities. We believe that this local DSB density, which we refer to as the cluster density, can be used as a predictor of chromosome aberrations. The residual DSBs at 24 hours of repair are also investigated. Our model predicts a constant fraction of DSBs left unrepaired, regardless of ion type, dose, or LET. From analysis of the results, we have developed correlations that are capable of predicting the yield of residual and misrepaired DSBs for our model. These correlations express the impact that the physics of the beam, dose and LET, has on the early biological effect, misrepaired and residual DSBs at 24 hours. The trends displayed by these correlations for misrepair and residual DSBs have a relevance to proton therapy planning.

It is recognised that the work contained here covers a wide range of fields which not all readers may be familiar with. We include a table summarising the field specific terms used (Supplement 1).

## Methods

### Simulation of DSBs

This work uses the Monte Carlo based toolkit Geant4 (10.02 patch 01)^38^, with the default G4EmDNAPhysics list. Incerti *et al*. have presented and validated the parameters of this physics list^39^, which has been used in a number of published DNA damage simulations^40–42^. The Geant4-DNA extension accurately models event-by-event particle tracking down to low energies, simulating each track as a series of steps determined by physical interactions.

We use this toolkit to simulate the transport of different ion species across a water medium representing a simplified cell model. Using water as a surrogate for biological material has become a standard assumption in Monte Carlo studies of DNA damage^43^. The cell geometry consists of a 5 µm diameter spherical nucleus, as might be typical of a lymphocyte^44^, in the centre of a 10 µm box, used as a surrogate for the cellular cytoplasm. A uniform average dose is delivered to the cell through methods described in Supplement 2. DSBs are calculated according to the principles of nanodosimetry through assessing the clustering of energy depositions within the nucleus. This work follows the methodology of Francis *et al*.^45^, where assumptions are made in order to convert energy depositions into strand breaks. The methodology assumes that a fraction of the nucleus is occupied by the sensitive material (DNA backbones). We determined the sensitive fraction of the nucleus as 15% by fitting the predicted initial yield of DSBs to literature data^40,42,46–48^, Supplement 3. The cell model implicitly considers indirect damage by fitting the sensitive fraction to literature data that includes indirect effects. In our model energy depositions in the sensitive volume above 37.5 eV are guaranteed to cause strand damage, whilst energy depositions below 5 eV cannot cause strand damage, with the probability increasing linearly across this range^46^. An accepted damage site is randomly assigned to strand 1 or 2 of the double helix and the position is recorded. Following the simulation of all primary particles, to achieve the required dose, the list of damage sites is analysed by a clustering algorithm based on a modified implementation of the Density-Based Spatial Clustering of Applications with Noise (DBSCAN)^49^ algorithm. The algorithm determines DSBs by clustering damage sites that are on opposite strands and separated by a maximum distance of 3.32 nm, equivalent to the separation of 1 helical turn (10 bp)^50^.

A separate simulation of the chromatin fibre is used to determine the structure of individual DSBs, most of the details of which were reported in our previous work^51^. We build a model of the double helix, with backbones and bases modelled as quarter cylinders and half cylinders^52^ with volumes of 0.28 nm^3^ and 0.13 nm^3^ respectively. The double helix is wound around cylindrical histones in 1.65 left-handed turns to form the nucleosome, which are then arranged in the solenoid chromatin conformation^53^. The chromatin is irradiated with primary ions matching the energy of those used in the cell model. For each incident ion, energy depositions are cumulatively scored in the DNA volumes and the same energy dependent probability of damage induction is applied (5–37.5 eV). Indirect damage is included with use of the Geant4-DNA chemistry modules^54^. Hydroxyl radicals crossing a DNA backbone or base are assigned a probability of inducing damage, with the probability fitted to produce 65% strand damage due to indirect effects when the fibre is irradiated by a 60-Co source^46^. DNA volumes damaged by direct and/or indirect effects are recorded per primary particle. The damaged volumes are analysed by an improved clustering algorithm, since both the genomic separation between damage sites and their strand is known. This removes any discrepancy that may arise due to damaged backbones within 3.32 nm but separated by more than 10 bp. A damaged base is included in the cluster if it is between 3 bp of the extreme ends of the backbones involved in the break^11^. Damaged bases directly attached to a damaged backbone are not included in the cluster since it is assumed that these will be removed along with the backbone during repair. The process is repeated for at least 10^6^ primary ions recording details of the induced DSBs per primary to develop a break complexity library.

The results of the cell and fibre model are combined to give the positions of DSBs, calculated as the central coordinate of the damage sites in the cluster, and the break complexity, defined as the number of extra SSBs and base lesions included in the break. This damage data is then passed to our repair model.

### Non-Homologous End Joining Repair Model

The geometric distribution and complexity of DSBs are used to convert each DSB into two pseudo-molecules representing exposed DNA strand ends. Ends are allowed to move within the cell nucleus by sub-diffusive motion, where mean squared displacement scales with time as t^α^, with α < 1. This has been shown experimentally to better model DSB end mobility compared to Brownian motion^29^. We have assumed the continuous time random walk implementation of sub-diffusion^55^. In this method DSB ends are assigned a random waiting time drawn from an exponential distribution, during which they do not move. After this waiting time, the end is displaced in a random direction with length drawn from a Gaussian distribution, and a new waiting time is assigned. Any proposed step leaving the nuclear envelope is rejected and resampled.

Simultaneous to their motion, DSB ends undergo a series of stochastic time constant based state changes. Each change of state represents the recruitment and action of a particular protein or complex of proteins, implicitly assumed to be randomly distributed throughout the nucleus. Time constants are fitted to reproduce the known recruitment kinetics of these proteins or complexes of proteins. We have chosen to model only the canonical non-homologous end joining (c-NHEJ) process as it is computationally less intensive; the impact of this is discussed later. The c-NHEJ process has an agreed core group of proteins which bind sequentially during repair^56^. DSB ends are initially placed with no associated proteins. The ends then either change to an inhibited state or to a state with Ku70/80 attached. The inhibited state represents attachment of proteins from competing processes occupying the DSB end and prevent Ku70/80 from binding. Once a DSB end has recruited Ku70/80 it cannot freely dissociate back to a naked DSB end^57^, but must progress to a state with DNA-PKcs attached. Once a DSB end has recruited Ku70/80 and DNA-PKcs (DNA-PK complex) it cannot dissociate to a previous state^58^. Two ends in the DNA-PK complex state, that are within 25 nm, can react to form a long range synaptic complex^59^. In this complex DNA-PKcs can cross/auto-phosphorylate, and in turn phosphorylate Ku70/80^57^. The synaptic complex can either dissociate to two DSB ends with no attached proteins, or move to a short range synaptic complex state^59^. Dissociation occurs with a short time constant resulting in only transient formation of the long-range complex^59^. We have used this model to reproduce results of fluorescent recovery after photo-bleaching (FRAP) experiments reported in the literature^58^. Once in a short range synaptic complex state the repair pathway cleans all extra lesions associated to the break before final ligation can occur, with time constants taken from literature^26^. The final ligation step represents the actions of XLF, XRCC4, DNA-Ligase IV, and polymerases. The c-NHEJ repair model allows us to investigate correctly and incorrectly repaired DSBs, as well as the number of residual DSBs for a given repair time. The full details of this repair model will be published separately at a later date.

### Data availability

The datasets generated during and/or analysed during the current study are available from the corresponding author on reasonable request.

## Results

### Misrepair and LET

**Figure 1:**
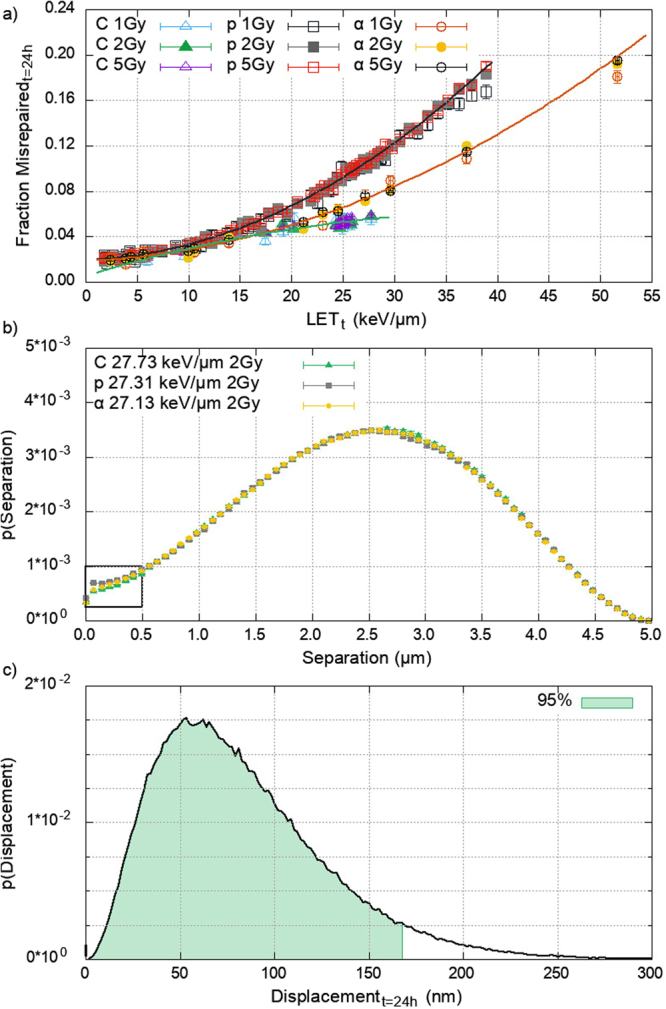
(**a**) The average misrepair of DSBs as a fraction of total DSBs joined at 24 hours at a range of LET_t_ for protons (p), alphas (α), and carbon-12^6+^ (C). A dose independent difference in misrepair between iso-LET_t_ ions is shown. Carbon-12^6+^ shows anomalous behaviour between LET_t_ of 20–30 keV/µm, discussed later. Error bars show the standard error in the mean between 200 repeat simulations. Lines show second order polynomial fits to guide the eye. (**b)** The DSB separation probability density function for iso-LET_t_ protons (p), alpha (α), and carbon-12^6+^ (C) in a 5 µm diameter spherical nucleus. The distributions show the probability that another DSB is located at a given separation from any DSB. Differences are only seen at small separations (inset), due to separations between intra-track DSBs. (**c)** The DSB end displacement at 24 hours. Showing that 95% of DSB ends move 168 nm or less in 24 hours. This limits DSB interactions to nearby neighbours where the DSB separation distribution is different between the ions.

The cell model was irradiated with 1, 2, or 5 Gy of protons, alphas, and carbon-12^6+^ across a range of track averaged linear energy transfer (LET_t_). The NHEJ model was run for a repair time of 24 hours, tracking motion, protein recruitment, and the repair of DSB ends. The fraction of DSBs that misrepaired was recorded, calculated as a fraction of the DSBs that are joined at 24 hours. Figure 1a shows a difference in misrepair between the three ions investigated, with the difference becoming more pronounced at higher values of LET_t_. Similar values for the fraction misrepaired are seen between different doses, though there is a difference in the absolute number misrepaired. Carbon-12^6+^ and alpha particles show relatively similar behaviour, however, protons have a higher fractional misrepair. We investigate this difference in terms of the repair pathway and the ion induced damage patterns.

Within the c-NHEJ model only two DNA-PK complexes can undergo synapsis, causing possible misrepair. Ku70/80 has a high affinity for naked DSB ends, and once loaded rapidly recruits DNA-PKcs^60–62^. The recruitment of Ku70/80 is largely unaffected by break complexity^62^, and both Ku70/80 and DNA-PKcs are abundant in the cell making depletion of local concentrations near composite break sites unlikely^63^. Therefore, in the model there is a similar and rapid formation of DNA-PK complexes at break sites, regardless of the radiation quality used. As such this cannot explain the difference in misrepair observed.

Different particles at iso-LET_t_ produce different track structures as they pass through the nucleus. The resultant difference in the energy deposition pattern leads to differences in DSB complexity. To determine the influence of break complexity on misrepair for a single ion species, the repair model was rerun for two cases; one populated with only simple DSBs and one populated with only complex DSBs. The predicted misrepair fraction for either case is not different from the data in Fig. 1a (Supplement 4). Furthermore, we investigate the effect that break complexity has on our ability to differentiate misrepair between ions species, using the same cases as above. For the data in Fig. 1a, the differences in misrepair are statistically indistinguishable below an LET_t_ of 5 keV/µm. Neither case alters this value, showing that within our model the simulated break complexity does not impact misrepair.

A further implication of the different energy deposition patterns is a difference in the spatial distribution of the DSBs themselves. We assess proximity between DSBs by determining the probability that a DSB is within a radial distance from another DSB. Figure 1b shows the probability density functions (PDF) of DSB separation for the three ion species at an iso-LET_t_, equivalent to the distal edge of the proton SOBP (≈27 keV/µm). A non-zero probability for separations of 0 µm is shown since it includes DSBs that are separated between 0 and 10 nm. Similarities at larger DSB separations are seen, indicating that it is equally probable to have DSBs separated by a value greater than around 0.5 µm. Below 0.5 µm a difference can be seen between the ions, due to separations of intra-track DSBs. Here, we see a greater probability of proximal DSBs in the proton-irradiated cell compared to the alpha and carbon-12^6+^ case. The importance of this scale is further established in Fig. 1c. This shows that the end displacement in 24 hours, 95% moving less than 168 nm, limits interactions to nearby breaks only and coincides with the DSB separations for which the iso-LET_t_ ions are different. As such break proximity can explain differences in misrepair.

### Cluster Density, Misrepair, and Residual DSBs

**Figure 2:**
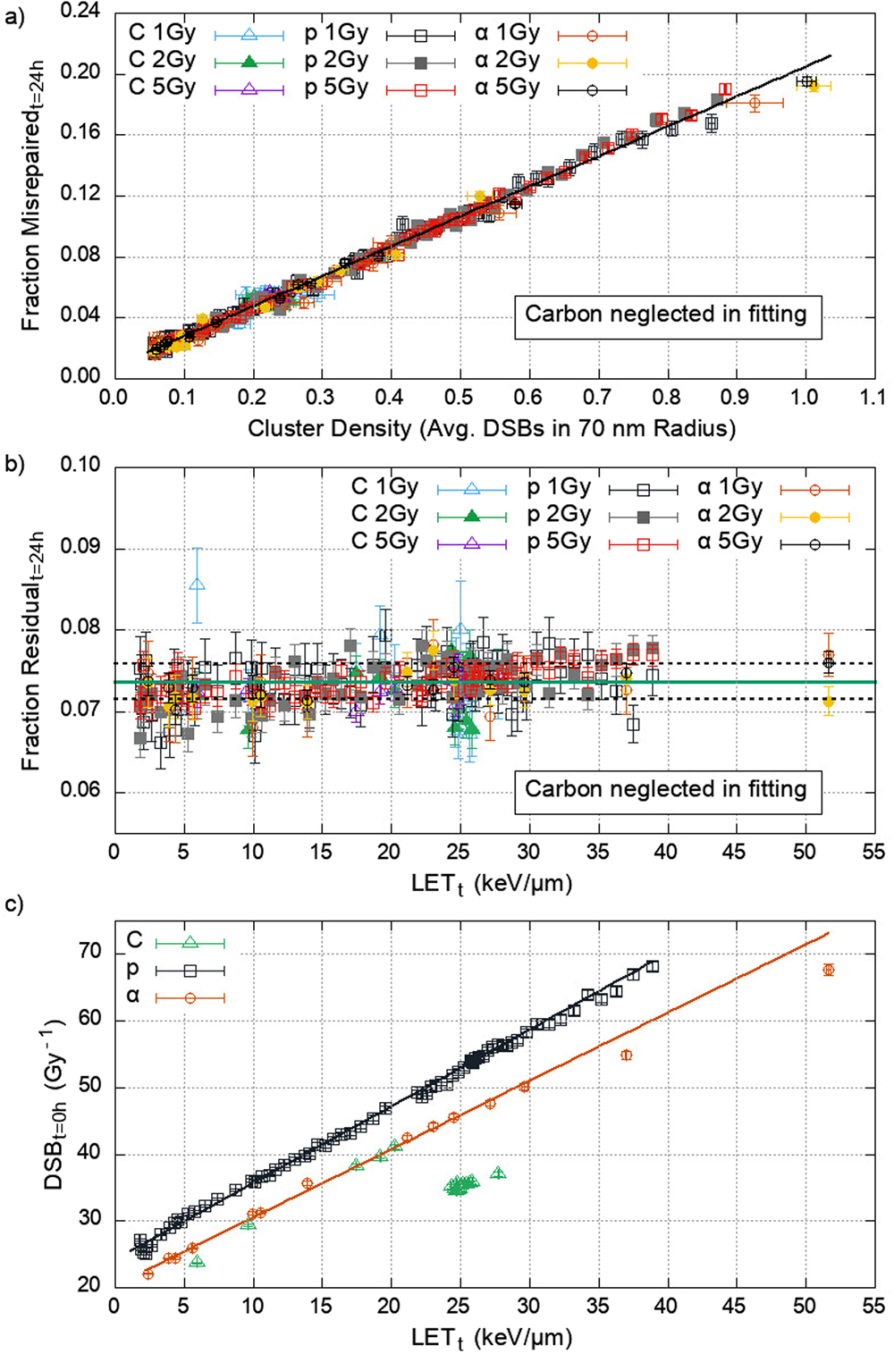
(**a**) The average number of DSBs separated by less than 70 nm from any given DSB (cluster density) and the corresponding misrepair at 24 hours for protons (p), alphas (α), and carbon-12^6+^ (C). Showing a linear relation (R^2^ > 0.9) with fitting parameters presented in Table 1. Error bars represent the standard error in the mean between multiple repeat simulations (2500 for cluster density, 200 for misrepair). (**b**) Fraction of DSBs left unrepaired following 24 hours of repair across the LET_t_ range investigated. The solid line shows a constant residual fraction of 0.073. Dashed lines show the standard deviation in this value. Error bars are the standard error in the mean for 200 repeats. (**c**) The predicted number of DSBs per unit dose for protons, alphas and carbon-12^6+^ as a function of LET_t_, with lines showing linear fits for protons and alphas. There is a clear discontinuity in carbon-12^6+^ data. Error bars are the standard error in the mean for 2500 repeats. Due to the anomalous behaviour of carbon-12^6+^, its data is not used to determine any correlations but is included in the plots for interest only.

To quantify the initial damage patterns, the average neighbouring DSBs within a given radius for each DSB is determined, which we refer to as the “cluster density”. If motion of DSB ends is predominantly responsible for misrepair, then this value scales with the number of potential incorrect partners a DSB end has available for interaction. The cell model was irradiated with the same radiation qualities as shown in Fig. 1a, and the average cluster density was calculated for 2500 repeat simulations. We find that the cluster density has the strongest correlation to misrepair when calculated with a radial distance of 70 nm, giving a minimum in χ^2^. However, the strength of this correlation is not greatly decreased by deviations of ±30 nm from this value (Supplement 5). Figure 2a shows a strong linear relation between cluster density and misrepair. This shows that the local DSB concentration (cluster density) in the initial damage pattern is predictive of the misrepair at 24 hours in our model. Based on our results, we suggest this physico-marker can be used to indicate the impact that beam parameters have on biological response. Cluster density can be estimated from the ion LET_t_ though a fitted second order polynomial (Supplement 6), with parameters given in Table 1. Generally, higher LET_t_ results in a higher cluster density, with protons producing a higher cluster density relative to iso-LET_t_ alphas (a similar trend as seen in Fig. 1a).

**Table 1:** The correlation fitting parameters developed in this work for protons and alphas with their asymptotic standard error shown as a percentage error.

Parameter	Proton ± %	Alpha ± %
a	0.1966 ± 0.4	0.1966 ± 0.6
b	0.008 ± 3.4	0.008 ± 3.4
c	0.0736 ± 0.2	0.0736 ± 0.2
d	1.149 ± 1.0	1.02 ± 2.5
e	24.10 ± 0.6	20.2 ± 1.3
f	4.879E-4 ± 0.8	3.25E-3 ± 5.0
g	2.84E-3 ± 4.7	1.61E-3 ± 33
h	5.13E-2 ± 1.6	5.51E-2 ± 5.7

In the context of radiotherapy, residual DSBs are another biological endpoint of interest. Our model predicts that the fraction of residual DSBs following 24 hours of repair is constant across the radiation qualities investigated, with an average of 7.3% breaks left unrepaired. Figure 2b shows that this constancy holds across the ion species, dose, and LET_t_ range investigated.

To apply the misrepair and residual correlations to a clinically relevant case, and to make predictions of absolute yields, the number of DSBs is determined as a function of LET_t_ and Dose. For the clinically relevant LET_t_ range of 0–20 keV/µm we observe a linear correlation to initial DSB yield for protons and alphas (Fig. 2c). This linear trend has been reported in the literature for both ions^46,48,64^. The DSB yield for alpha particles begins to deviate from linearity above ≈30 keV/µm. However, in the rest of this work we only use the correlation between the bounds of its linearity. The constant fraction of residual DSBs at 24 hours combined with the linear dependence of initial DSB yield on LET_t_ leads to a linear relationship between the yield of residuals and LET_t_ (Supplement 8); this has been experimentally observed for 53BP1 foci under similar conditions by Chaudhary *et al*.^65^, although at a systematically lower yield. Potential causes for this are discussed later. The parameters for all correlations are given in Table 1.

The DSB yield for carbon ions is linear up to ≈20 keV/µm, after which there appears to be a discontinuity, also noticed by other users of Geant4-DNA (personal communication S. McMahon). The behaviour originates from a switching between relativistic and classical calculations in the Geant4-DNA models. This may have implications for studies simulating clinical carbon ions (Supplement 7). It is possible to use only relativistic calculations, though this has not been validated by the Geant4-DNA collaboration, and further implications are unknown. As such we have chosen to use the default Geant4-DNA models and not include carbon-12^6+^ data in any of the correlations.

### Residual and Misrepaired DSB Yields

Here we present the correlations from Fig. 2a,b and c. We convert the correlations of fractional misrepair and residual DSBs into absolute yields, shown in equations (4) and (6). In order to apply the correlations to an example of a clinical proton SOBP, we determine the dependency on dose and LET_t_, shown in equations (5) and (7).

1 $${{F}}_{{Mis}{.}}={a}\cdot {Cluster}\,{Density}+{b}$$

2 $${{F}}_{{Res}{.}}={c}$$

3 $${{N}}_{{DSB}}={D}\cdot ({d}\cdot {L}+{e})$$

4 $${Misrepair}\,{Yield}={{N}}_{{DSB}}\times \{{a}\cdot {Cluster}\,{Density}+{b}\}\times \{1-{c}\}$$

5 $${D}\cdot ({d}\cdot {L}+{e})\times \{{a}\cdot [{f}\cdot {{L}}^{{2}}+{g}\cdot {L}+{h}]+{b}\}\times \{1-{c}\}$$

6 $${Residual}\,{Yield}={{N}}_{{DSB}}\times {c}$$

7 $${D}\cdot ({d}\cdot {L}+{e})\times {c}$$

Equations 1–7: F_res_ is the fraction of DSBs that are unresolved at 24 hours, F_mis_ is the fraction of DSBs that are misrepaired at 24 hours, N_DSB_ is the initial number of DSBs induced by the exposure, Cluster Density is the average number of neighbouring DSBs within a 70 nm radius, L is the track averaged linear energy transfer in units of keV/μm, D is the dose in units of Gy, and a-h are parameters determined through best fitting, the values of which are reported in Table 1 for protons and alphas.

**Figure 3:**
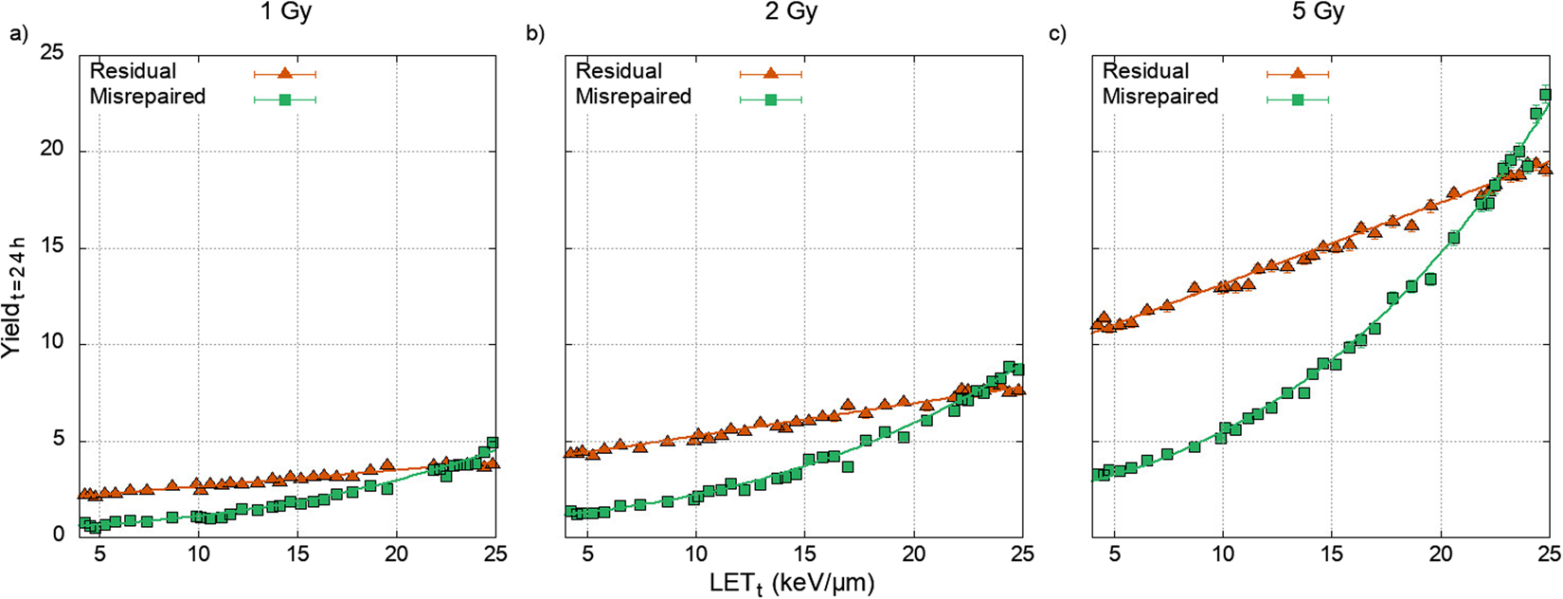
The correlation predicted yield of misrepaired, equation (5), and residual, equation (7), DSBs across the clinically relevant proton LET_t_ range, with a constant dose of (**a**) 1 Gy, (**b**) 2 Gy, and (**c**) 5 Gy. Points show values measured within the simulation. Error bars in the yield are the standard error in the mean of 200 repeats. Error bars in the LET_t_ are the standard error in the mean for 50,000 repeats. Both yield and LET_t_ error bars are too small to be seen on this scale.

Figure 3 shows how the yield of residual and misrepaired DSBs predicted by equations (5) and (7) increases across a clinically relevant LET_t_ range^65^ whilst keeping dose constant. The plotted data points show the results of our model and show good agreement with the correlations derived earlier. There is a difference in behaviour between the predicted yield of residual and misrepaired DSBs, with misrepaired DSBs becoming more important at high LET_t_.

**Figure 4:**
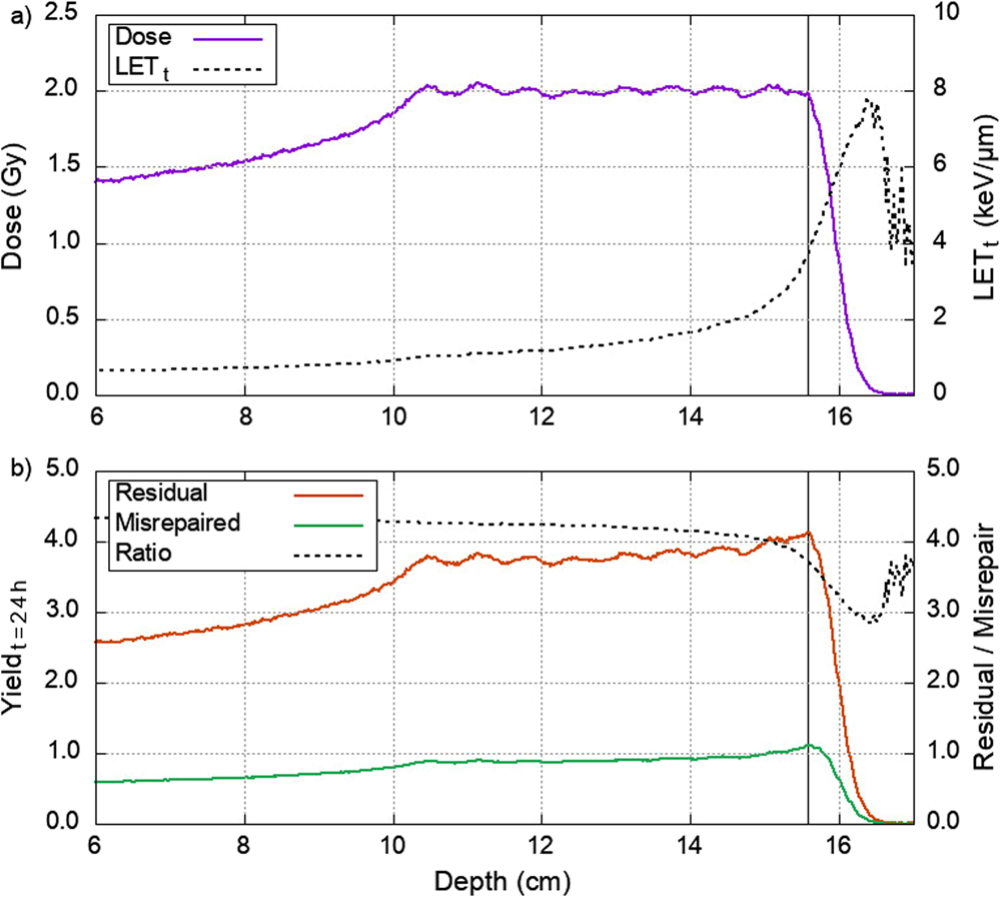
(**a**) The depth dose (solid) and LET_t_ (dashed) profile of an example proton SOBP with 2 Gy across the plateau comprised of nine pristine Bragg peaks. (**b**) The predictions of residual and misrepaired DSB yields for the same proton SOBP, calculated with equations (7) and (5) respectively. The ratio of residual DSBs to misrepaired DSBs is shown as a dashed line. The solid black vertical line on both graphs denotes the depth where the physical dose begins to fall off (15.58 cm).

The proton SOBP depth dose and LET_t_ profile is simulated using the Geant4 “QGSP_BIC” physics list (Fig. 4a). Here, the SOBP is simulated from the combination of nine pristine Bragg peaks, where the maximum proton energy is 150 MeV. Using equations (5) and (7), the expected yields of misrepaired and residual DSBs per nucleus is calculated with proton depth (Fig. 4b). Due to low proton fluence there is increasing noise in LET_t_ at the distal end of Fig. 4a. However, since the dose in this region is negligible the noise in LET_t_ doesn’t impact our predictions. The undulation of the plateau is due to combination of the 9 pristine Bragg peaks. Figure 4b shows a gradual increase in residual and misrepaired DSBs across the SOBP, with a more pronounced peak at the distal end. After this, the yields of residual and misrepaired DSBs fall off at a slower rate than physical dose, resulting in biological effect at low dose regions past the SOBP. Interestingly, the peak of residual DSBs coincides with the distal edge of the dose plateau whilst the peak of misrepaired DSBs is situated at a slightly increased depth. Combined with Fig. 3 this demonstrates the higher sensitivity that misrepair events have to LET_t_. For this 2 Gy SOBP misrepaired DSBs are always predicted at a lower yield than residual DSBs; however, the plotted ratio shows that the relative importance of misrepair is highest at the distal edge.

## Discussion

In this work, we demonstrate plausible mechanisms which, combined, lead to the conclusion that the early biological effect of radiation depends on the extent to which DSBs are clustered, shown with the cluster density.

The fraction of misrepaired DSBs can be predicted by cluster density; independent of ion type, dose, LET_t_, and break complexity. The cluster density produced by different ion species is not the same for the same LET values (Fig. 1b), and therefore produce different early biological effect (Fig. 1a). We suggest that this can explain the experimentally observed differences in cell kill and mutation caused by different ion species at iso-LET^35^. This cluster density dependent response is the result of three factors: the time required for an exposed DNA end to be prepared for re-joining, the motion of these broken ends during this time, and the fact that formation of synaptic complexes depends on co-localisation of two DSB ends. The diffusion of breaks will likely result in separation of initially co-localised correct partners and, in regions of high DSB density, increases the possibility of co-localising with an incorrect partner. We quantify this in terms of cluster density. The cluster density can be estimated from LET_t_ for a given ion type (Supplement 6) and is dose independent. Only intra-track DSBs are proximal enough to contribute to the cluster density. We do not see any considerable numbers of DSBs formed as a result of inter-track energy depositions, which we have investigated up to 5 Gy (data not shown).

Our model predicts that the fraction of DSBs left unrepaired is constant; independent of ion type, dose, and LET_t_ (Fig. 2b). This arises due to the tight confinement of DSB ends around their initial break site, Fig. 1c. Only a small fraction of DSB ends are able to escape this confinement, and are then unlikely to meet another end. The escaping fraction is solely a product of the sub-diffusive motion and therefore not dependent on the beam parameters. The number of initial breaks has a linear relationship with dose and with LET_t_, we therefore predict that the absolute yield of residual DSBs scales linearly with these factors. This is in agreement with current clinical practice, where treatment plans use dose as a surrogate for cell kill. Furthermore, it lends weight to the concept of LET optimisation.

Individual NHEJ pathways are thought to respond to different break complexities^66^. Our repair model only contains the DNA-PKcs dependent c-NHEJ pathway, capable of resolving simple and complex breaks. For this reason the kinetics of our model were fitted to experimental data from laser generated complex DSBs^58^. The model omits DNA-PKcs independent pathways, which resolve simple breaks and have been suggested to complete faster^66^. However, the above mechanism of misrepair is still applicable for these pathways, since they also require time dependent recruitment of proteins to mobile ends before synapsis between co-localised partners. Differences would arise in the time required to prepare ends for re-joining, leading to a difference in the radius at which the cluster density should be determined. This means that our cluster density radius is likely an misestimation for fully NHEJ capable cells. The effect of this is somewhat dampened by the fact that some proportion of these simple DSBs can be resolved by c-NHEJ. We therefore do not expect the addition of DNA-PKcs independent repair pathways to substantially impact the results presented.

The recruitment kinetics of Ku70/80 and DNA-PKcs are fitted to laser irradiated *in vitro* Xrs6 and V3 cells with c-NHEJ pathways^58^. Repair kinetics are fitted to proton irradiated *in vitro* AG01522 cells^65^. As such the response of our model is not representative of a specific cell type. However, it can be shown that the mechanism of cluster density dependent misrepair holds for any NHEJ capable cell type, following the same justification as above. The induction of DNA damage in this model is fitted to the DSB yields of other *in silico* models for a range of cell types^40,42,46–48^, with a constant nucleus size of 5 µm diameter. Therefore, any deviation from this size will change the DSB density, as the same number of DSBs will be distributed across a different volume. The initial damage is therefore also not representative of any specific cell type. A misestimation in the initial DSB yield would lead to misestimation in the yields of residual and misrepaired DSBs. A misestimation in the DSB density would lead to changes in the cluster density, and consequently in the fraction of misrepaired DSBs. However, potential misestimation only changes the initial yield, not the mechanism of cluster density dependent misrepair. Given all of the above, we would expect the correlations detailed in this work to hold for both end points in any NHEJ capable cell, but with cell specific changes in the predicted yields. This change in yield, but not in trends, could explain the systematically lower yield of residual DSBs compared to the experimental results of Chaudhary *et al*.^65^.

The model does not include the Homologous Recombination (HR) pathway which is an “error free” pathway available to cells occupying the relevant cell cycle stages. It has been shown that NHEJ is a faster repair pathway than HR^67^, and is dominant throughout the cell cycle^67^. The core c-NHEJ proteins, DNA-PKcs and Ku70/80, have similar, rapid, recruitment kinetics throughout the cell cycle^58^. Phosphorylation of these recruited proteins causes dissociation from DSB ends, in our model this has a time constant of 95 seconds, providing an opportunity for processing by HR^58^. However, our model shows that the timescale of interest for predicting misrepair is much less than this (Supplement 9) and therefore, we do not expect addition of HR to cause a notable deviation in cellular behaviour from current predictions.

We have implemented sub-diffusion in our model by a continuous time random walk method with no form of tethering. This results in limited motion of DSB ends on the order of 100 nm at 24 hours, similar to that which has been reported for live cell experiments^28^. This limited motion means that the size of the nucleus, on the order of microns, does not affect the availability of potential partners. In V(D)J recombination the sub-diffusive motion is better described by fractional Langevin motion (fLm)^68^. If fLm were to apply to DSB repair in general, broken ends would be more strictly confined around their formation site. However, the confinement scale proposed would allow for an exploration volume that is larger than the volume of interest for misrepair, which we show to have a radius of around 70 nm (Fig. 2a). Therefore, we do not expect it to influence misrepair substantially.

The scale of motion associated with DSB ends *in vivo* is a debated topic within the literature. The only assumption made regarding this in our model is that the motion is sub-diffusive. The magnitude of the mobility of these ends was then scaled in order to reproduce the endpoint of residual breaks reported in literature^65^. The final scale of motion, and the fact that it agrees with the more conservative experimental results, is therefore an emergent property. To achieve greater mobility whilst retaining agreement to the literature reported residual yields would require a different mechanism. One possible mechanism that has previously been proposed, and observed experimentally by some^33^, is the formation of repair centres. This directed motion could result in large displacements of DSB ends whilst still maintaining their proximity, a necessary component for resolution of breaks.

This work uses a detailed short segment of the chromatin fibre to determine break complexity. The model includes damage from both direct and indirect effects in order to predict the DSB complexity. However, we do not consider the impact of some chemical effects such as the oxygen fixation hypothesis^69,70^. Inclusion of these processes would likely yield higher residuals at 24 hours due to a decrease in repair efficacy. Furthermore, our process of converting damage sites into DSBs is a simplification that does not account for deletions of very short DNA segments from complex breaks. If these segments are of insufficient length to recruit repair proteins^71^ they are both irreparable and experimentally unmeasurable in fluorescent repair kinetics experiments^72^. We therefore would expect to underestimate the true yield of residuals.

We have discussed reasons that lead us to believe the mechanisms behind residual and misrepaired DSBs are applicable to all NHEJ capable cells. This would result in conserved behaviour between cell types, but with cell specific yields of residuals and misrepaired DSBs. We therefore propose that the general behaviour predicted by our model from the interaction of these mechanisms is representative of reality.

The components of the biological response, residual and misrepaired DSBs, are correlated to our results and combined to give equations (4) and (6). These correlations show how the physical descriptors of the incident beam that are relevant to biological response can be summed up using cluster density. The mechanisms we have proposed for misrepair and residuals respond differently to LET_t_, Fig. 3, however both scale equally with dose. This means that our model predicts no change in the ratio of cells undergoing misrepair for either hypo or hyper fractionation. However, it also means that regardless of dose, misrepair will be dominant at LET_t_ corresponding to the dose fall-off region of the proton SOBP, >22 keV/µm, Fig. 3b. Assuming that misrepair is a marker for chromosome aberration this potentially means areas of higher genomic instability are created at the distal edge of the treatment volume. A second clinically relevant trend is predicted; using the 2 Gy case as an example, Fig. 3b, a change in LET_t_ from 20 keV/µm to 10 keV/µm reduces the average yield of misrepaired DSBs from 6.1 to 2.1, whilst only decreasing the average yield of residual DSBs from 6.8 to 5.1. Treatment plans extend the prescribed dose region beyond the gross tumour volume (GTV) partly in order to treat microscopic spread. In these regions, and bordering areas, it would seem that induction of genomic instability could be limited by moving high LET components into the tumour, whilst a similar cell kill potential could be achieved by maintaining dose.

Predictions of our model can be derived from more conventionally scored parameters in proton therapy, equations (5) and (7). The predicted biological response, shown in Fig. 4, follows similar trends to the phenomenological predictions of RBE across a proton SOBP^9,73^. In both cases there is a gradual increase in response across the dose plateau with a pronounced peak at the distal edge. As such it lends weight to the concept that RBE is driven by a combination of residual and misrepaired DBSs. Due to both residual and misrepaired DSB yields falling off at a slower rate than physical dose, the model predicts considerable biological effect past the distal edge of the SOBP. Misrepair falls off at a slower rate than residual DSBs, however the latter still dominates the yield. Therefore, we suggest that the biologically extended range reported by Marshall *et al*.^9^, and others, is most likely due to the presence of residual DSBs. For the proton SOBP presented here, misrepaired DSBs are predicted to peak at a slightly increased depth compared to residual DSBs, although the yields always remain lower. During dose escalation, this peak will be the first region where misrepair starts to dominate the biological response. Since the misrepair peak is deeper than the physical dose peak, this could have implications clinically.

Methods of LET optimisation have been suggested for clinical use, with the justification that RBE is dependent on LET^73–75^. The difference in response of residual and misrepaired DSBs to LET_t_ further emphasises the benefits of this type of planning, but suggests that prediction of biological outcome is not a straightforward combination of dose and LET. Therefore, if the benefits of proton therapy are to be fully exploited, the mechanisms we have proposed should be considered at the treatment planning stage. This could allow the manipulation of not only RBE in general but also the dominant pathway taken to cell death; a difference which could possibly be exploited clinically.

## Electronic supplementary material

Supplementary Information
