# Supplementary material for: In Silico Non-Homologous End Joining Following Ion Induced DNA Double Strand Breaks Predicts That Repair Fidelity Depends on Break Density
Source: Sci Rep. 2018 Feb 8;8:2654. doi: 10.1038/s41598-018-21111-8 (PMC5805743; doi:10.1038/s41598-018-21111-8)
Supplement: Supplementary file 1 — Supplementary Information [file 41598_2018_21111_MOESM1_ESM.pdf]

In Silico Non-Homologous End Joining Following Ion Induced DNA Double Strand Breaks Predicts That Repair Fidelity Depends on Break Density

N T Henthorn[1, *], J W Warmenhoven[1, *], M Sotiropoulos[1], R I Mackay[2], N F Kirkby[1,3], K J Kirkby[1, 3] and M J Merchant[1, 3]

[1] Division of Molecular and Clinical Cancer Sciences, Faculty of Biology, Medicine and Health, University of Manchester, UK

[2] Christie Medical Physics and Engineering, The Christie NHS Foundation Trust, Manchester, UK

[3] The Christie NHS Foundation Trust, Manchester, UK

[*] Both authors contributed equally to this work

Supplementary Information

1. Field specific nomenclature

This multi-disciplinary work covers a broad range of topics. To aid the reader we have included a table defining some terms that not all readers may be familiar with.

Primary particle	The initial incident ion that traverses the target
Linear Energy Transfer (LET)	The rate of energy deposition per unit length. Given in units of keV/µm.
Track averaged LET	The LET measured and averaged across a number of mono-energetic ions.
Radiation quality	The collected properties of the radiation used; including parameters such as LET, species, etc.
Nanodosimetry	A dosimetric technique that measures energy depositions on a similar length scale to the structure of DNA, i.e. at the nano-scale.
Pristine Bragg peak	The profile of energy vs position for a mono-energetic fixed beam of particles.
Spread Out Bragg Peak (SOBP)	A series of pristine Bragg peaks, at different energies, that are weighted in order to produce a smooth plateau of high dose across a target.
Fractionation	A dose of radiation is often prescribed in fractions in order to give healthy tissue a chance to repair. A typical course of radiotherapy will normally be 40-60 Gy given in doses of 1.5-2 Gy per day.
Hypo/Hyper fractionation	Hypo fractionation uses less exposures of higher dose while hyper fractionation has more doses each consisting of a lower dose (although more than 1 dose may be delivered on the same day).
Direct/Indirect damage	Direct damage refers to DNA damage caused by the physical processes of the interaction of the beam. Indirect damage results from indirect or secondary interaction with the beam, i.e. when a free radical formed by the beam attacks the DNA.
Lesion	A site where the DNA structure is damaged.
Aberration	Successful repair of damaged DNA that has paired incorrect partners, resulting in a mixup of the genetic code.

Cluster Density	A measure of the average local DSB density in a cell nucleus.
alpha/beta	A measure that gives an indication of how sensitive a cell is to radiation. This ratio is derived from the linear quadratic model of cell survival.
Synapsis	The joining together of two exposed ends of the DNA helix.
V(D)J recombination	A process in the immune system where double strand breaks are purposefully induced and fixed in different combinations to increase the diversity of antibodies etc.
intra/inter track	Intra track refers to events created along a single ion track. Inter track refers to events from different ion tracks.
Monte Carlo	The method of simulation through repeated random sampling.
Sub-diffusion	Diffusion where the mean squared displacement does not scale linearly with time, resulting in the object being more spatially confined.
Fractional Langevin Motion	A form of sub-diffusive motion where the object is confined by a visco-elastic boundary.

Table S1. Definitions of some field specific terms.

2. LET and dose calculation

The track averaged linear energy transfer (LET_t) of the particle is determined through a separate Geant4-DNA simulation. A particle of a given energy is simulated crossing a water box of side length 10 µm, corresponding to the distance travelled across the cell nucleus and cytoplasm. Upon entering the box the particles coordinates and energy are recorded. The position and energy are then recorded for the particles final step within the volume. The LET_t is calculated as the change in energy divided by the distance travelled (assuming a straight path between the first and final step). Secondary electrons are "killed" within the simulation, this assumes that all secondary energy is deposited locally (unrestricted LET). The process is repeated for 50,000 primaries. The calculated LET_t for each of the 50,000 primaries forms an LET_t distribution, shown for some of the proton, alpha, and carbon-12^{6+} energies as the normalised distribution:

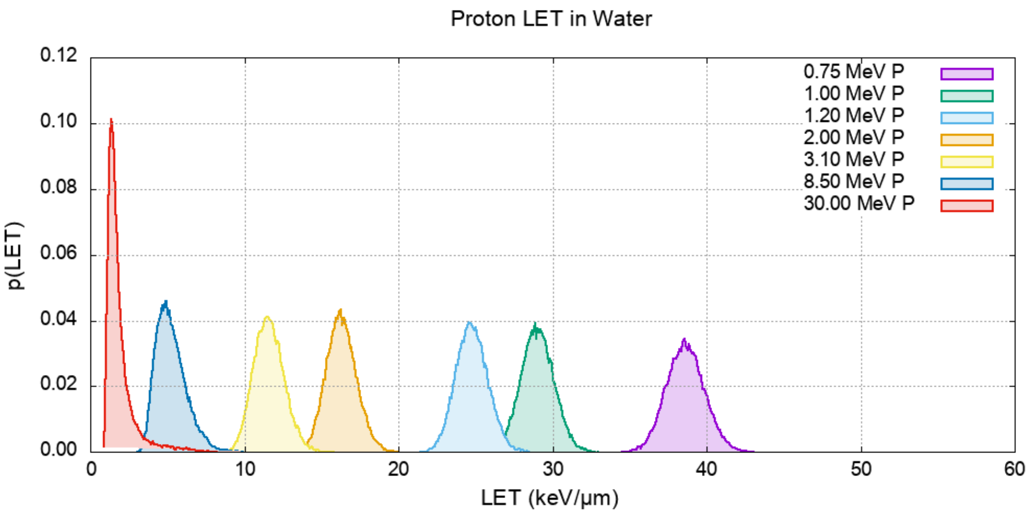

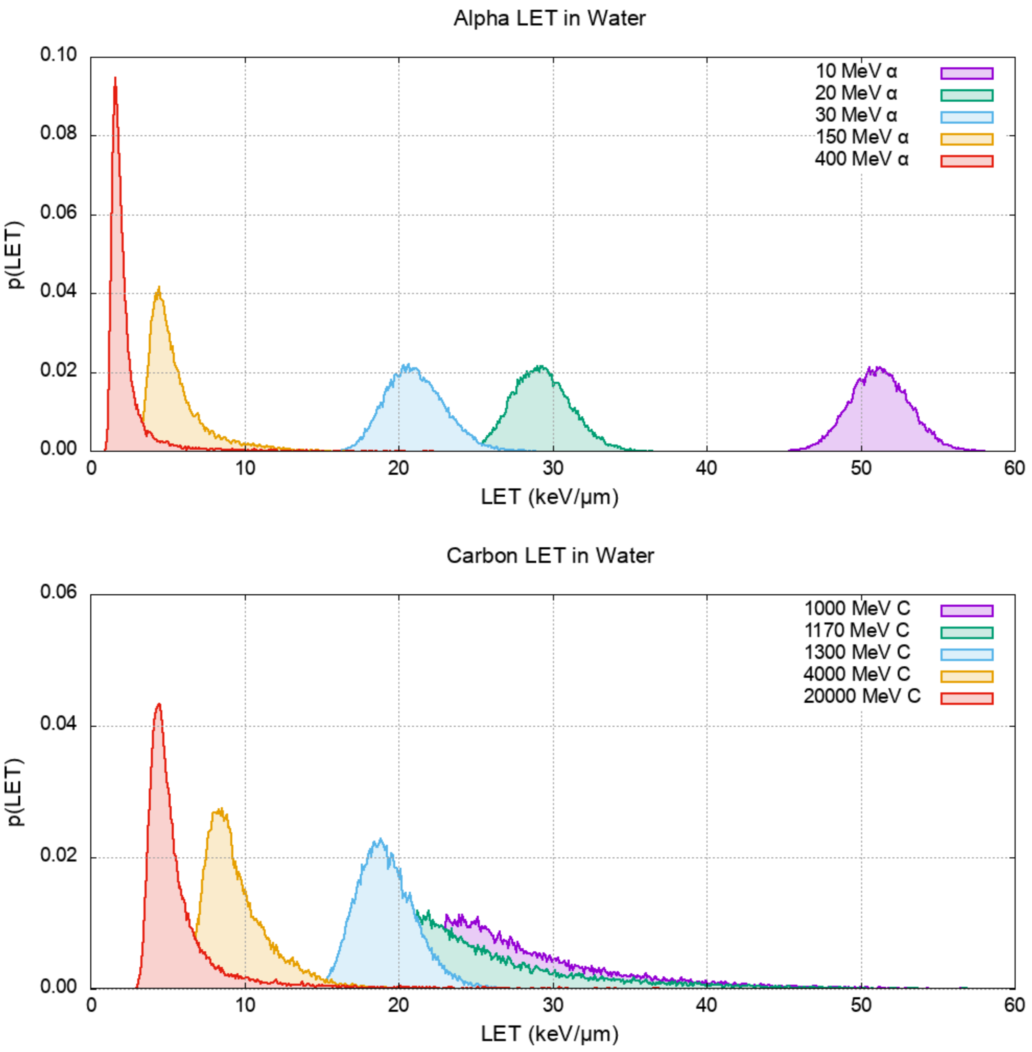

Figure S1. The probability distribution functions for the LET of mono energetic protons, alphas, and carbon ions. Higher energy particles have lower LET and vice-versa.

The average LET_t is calculated for each of the primary energies, with the standard error in the mean taken as the uncertainty. This gives the track averaged LET_t across the irradiation volume. This is shown for protons, alphas, and carbons for the energy range used in this work:

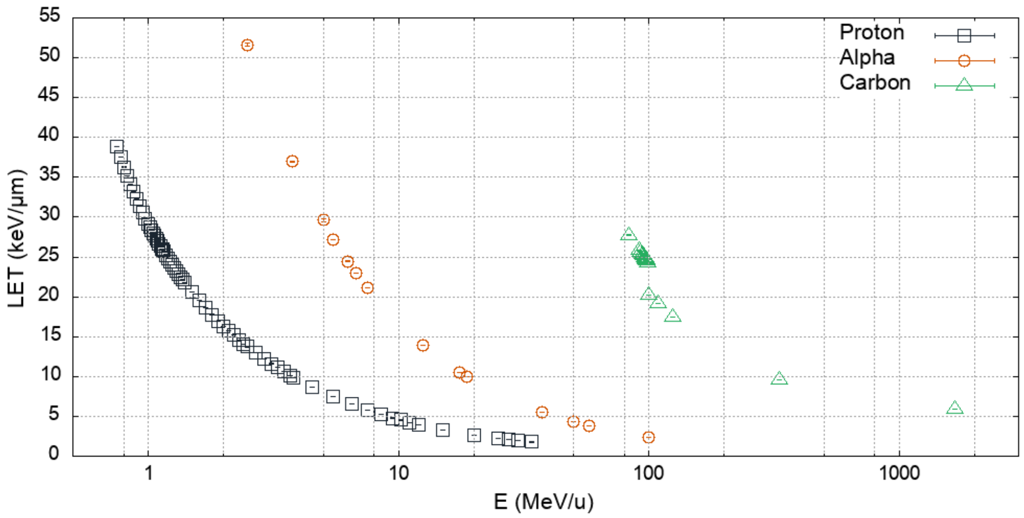

Figure S2. The average LET of the mono energetic ions simulated across a 10 µm thick water phantom.

The average energy deposited by a primary, of a given energy, crossing the 10 µm box is also recorded. The deposited energy is converted to a dose by dividing by the mass of the irradiated volume. In this work, a primary starts on a disc of radius equal to the cell nucleus (2.5 µm). This gives a cylindrical irradiation volume, with length of 10 µm (the nucleus and cytoplasm). Within the simulation the volumes are constructed of liquid water, giving an irradiated mass of 1.96E-13 kg. The dose per primary for protons, alphas, and carbons in the energy range used in this work is shown as a function of the primary LET_t across the nucleus:

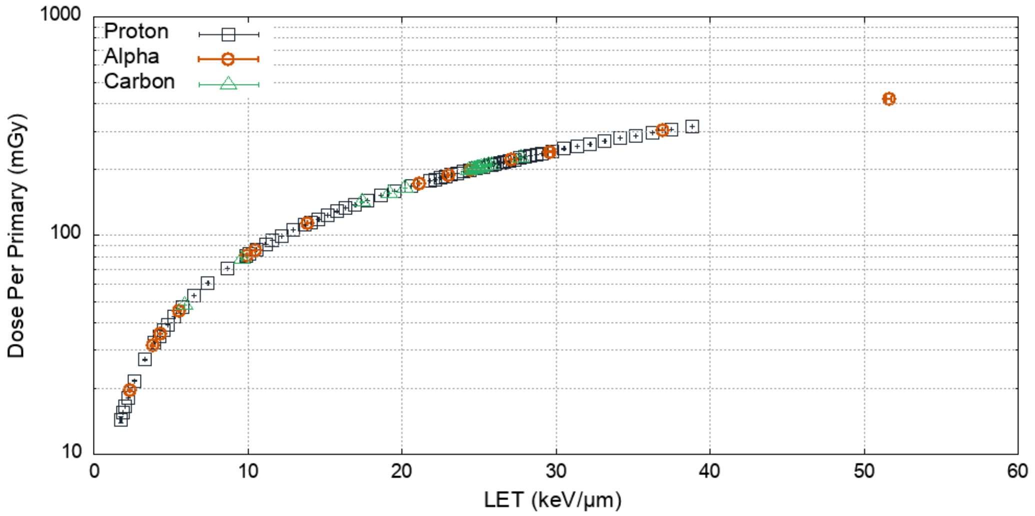

Figure S3. The dose delivered to the cell nucleus per primary, showing higher dose depositions from high LET primaries.

Using the dose per primary data the number of primaries required to deliver a given dose is calculated. However, this often results in non-integer values. To overcome this the irradiation field is overextended. Initially the primary is randomly placed on a disc with radius equal to the cell nucleus (2.5 µm). This ensures that every particle simulated crosses the nucleus, maximising computing resources. By increasing the disc radius and the number of primaries it is possible to average the number of primaries crossing the nucleus, and therefore give an average dose to the cylinder containing the nucleus. For example, an irradiation disc, r_{disc}, can be set up so that of the initial primaries, y, only an amount of them, x,

fall within the disc corresponding to the nuclear radius, r_{nuc}. Here x is equal to the number of primaries required for a given dose. This is shown schematically:

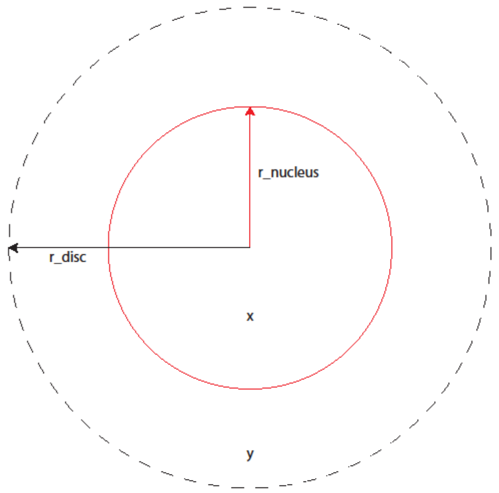

$$r_{disc} = \sqrt{\frac{y}{x} r_{nucleus}^2}$$

Figure S4. Schematic of the irradiation setup. Primary particles originate from a disc (dashed circle) with random coordinates. The radius (r_disc) is changed so that on average x particles originate in the disc covering the nucleus (red circle).

Where y is an integer value representing the number of primaries used, taken here as four times the required primaries, x, rounded to the nearest integer. This results in a slight under- or over-dosing for a single simulation, however, the effect averages out over multiple simulations.

The ion range is determined across a water phantom, shown for some of the primary proton and alpha energies. For the carbon ion energies investigated the range was greater than the water phantom length (1000 µm).

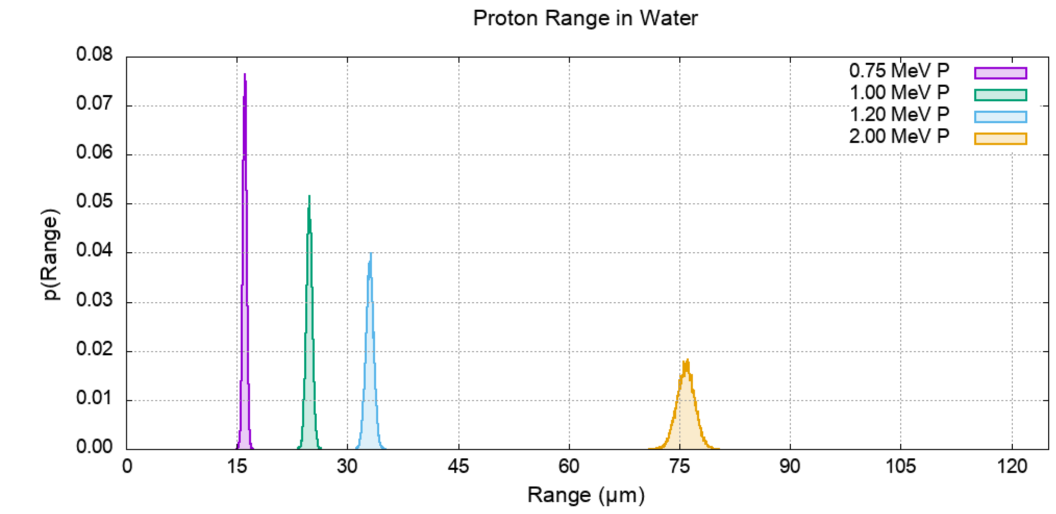

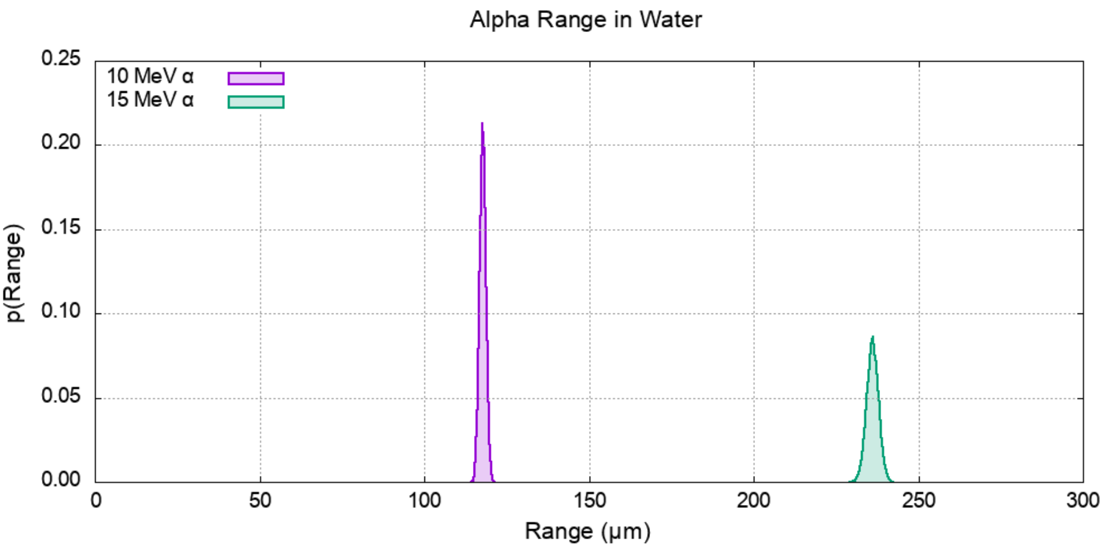

Figure S5. The simulated range distribution of mono energetic protons and alphas crossing liquid water. Range is measured as the position of the final simulation step made by the ion. All energy carbon ions investigated had a range greater than 1000 μm.

3. Predicted DSB yield compared to literature data

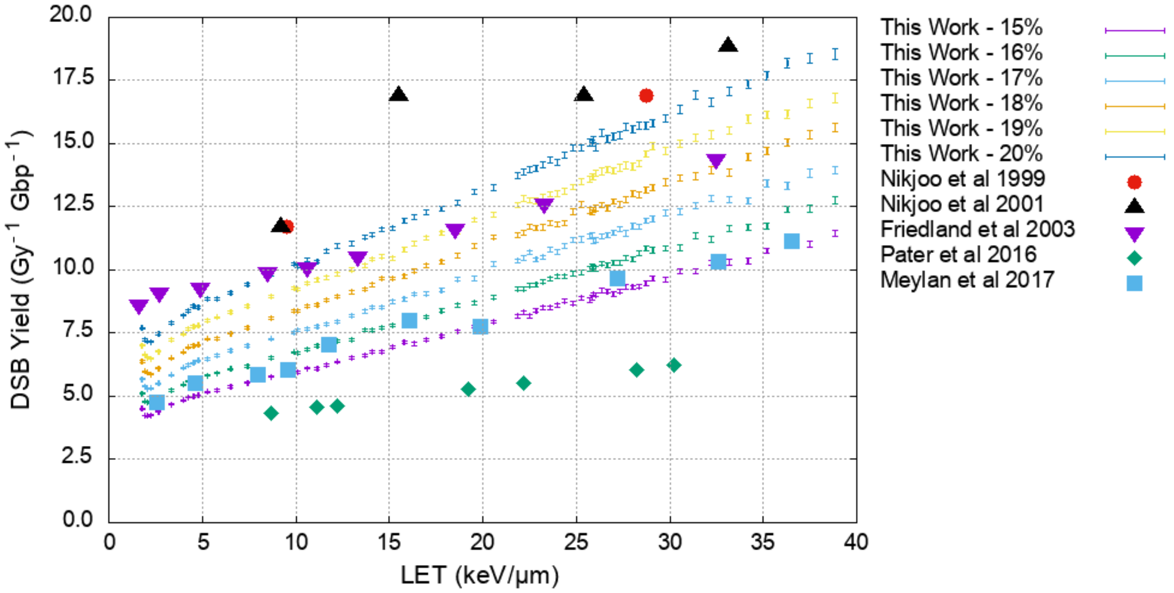

Figure S6. The predicted DSB yield across the LET range. Yields are shown for a range of sensitive nucleus fractions (15%-20%). 15% reproduces the yields reported by Meylan *et al.*

The DSB yield simulated with our model varying the sensitive percentage of the cell nucleus between 15 - 20%, assuming that the genome consists of 6 Gbp. Error bars show the standard error in the mean for LET_t (50,000 repeats) and the DSB yield (2,500 repeats). The DSB yields are compared to yields reported by other simulations in the literature[40–44]. Selecting a 15% of the nucleus as sensitive reproduces the reported DSB yields of Meylan *et al*[44]. The simulation of Meylan *et al* consists of a detailed fibroblast model including DNA damage from both direct and indirect effects, and is able to reproduce experimental yields of DSB induction.

4. Damage complexity and misrepair

To investigate the effect of damage complexity on the predicted misrepair the complexity is forced as either "simple" or "complex". For the "simple" case each DSB is placed in the repair simulation as only two damaged backbones. For the "complex" case the most complex break is selected from the damage library. Figure S7 shows the effect of "simple" or "complex" breaks on the fraction of misrepaired DSBs. Here, we do not see any significant change in the misrepair from either case.

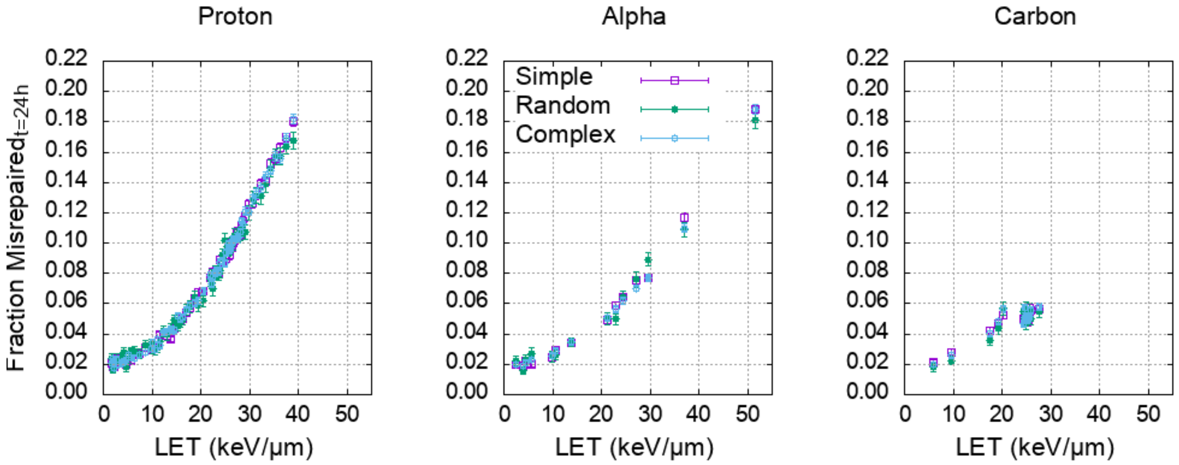

Figure S7. The predicted DSB misrepair when each DSB is populated by the simple or complex form. Random shows the case of randomly selecting the complexity from the break library. No significant differences are seen between the cases. Error bars show the standard error in the mean.

5. Fit of Cluster Density and Misrepair

Linear fits between the cluster density and misrepair, calculated with different radii, R.

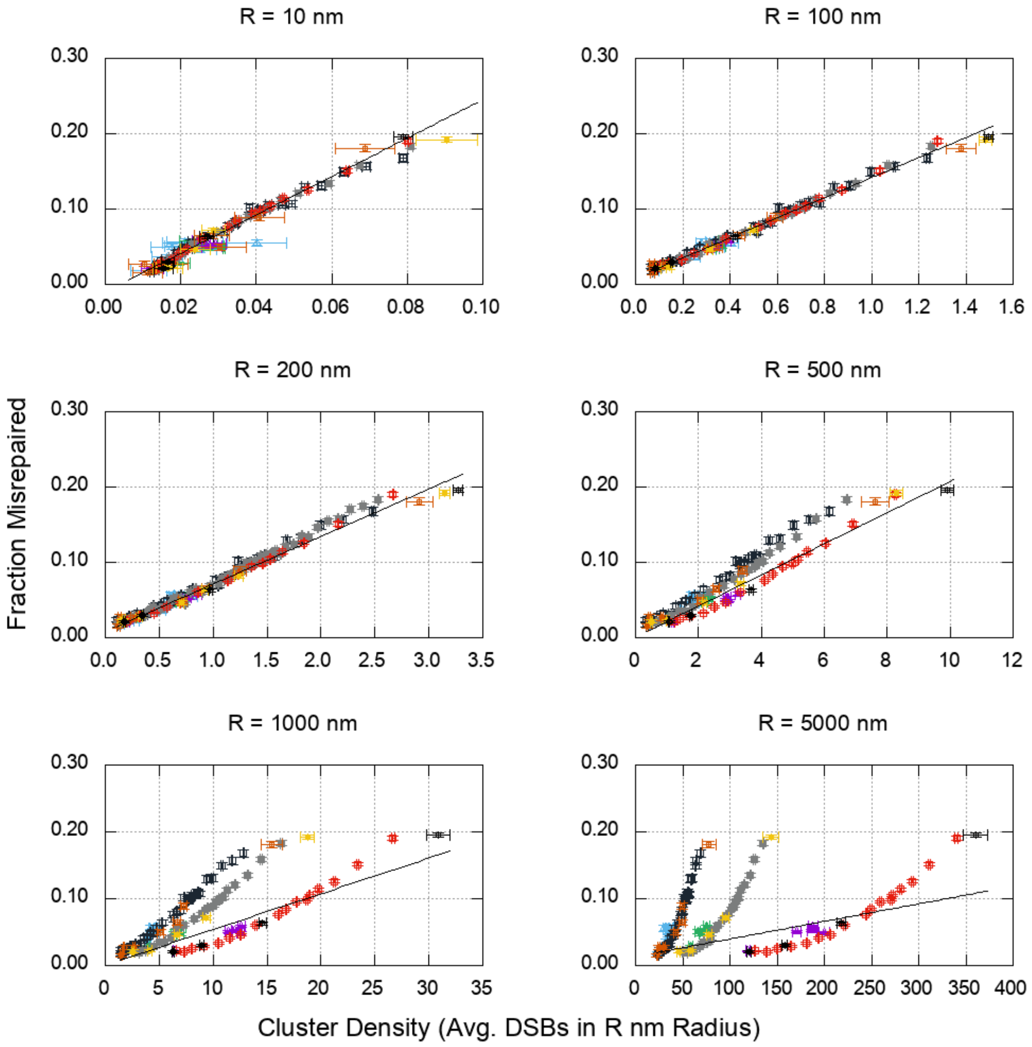

Figure S8. The average number of neighbouring DSBs calculated for a range of radii against the fraction of misrepair. Linear fits to the complete data set are shown by solid lines.

The goodness of the linear fit at each radii is calculated as the Pearson chi square. The goodness of fit is normalised to the maximum chi squared, showing a minimum at 70 nm. Though, chi square values are similar below 100 nm.

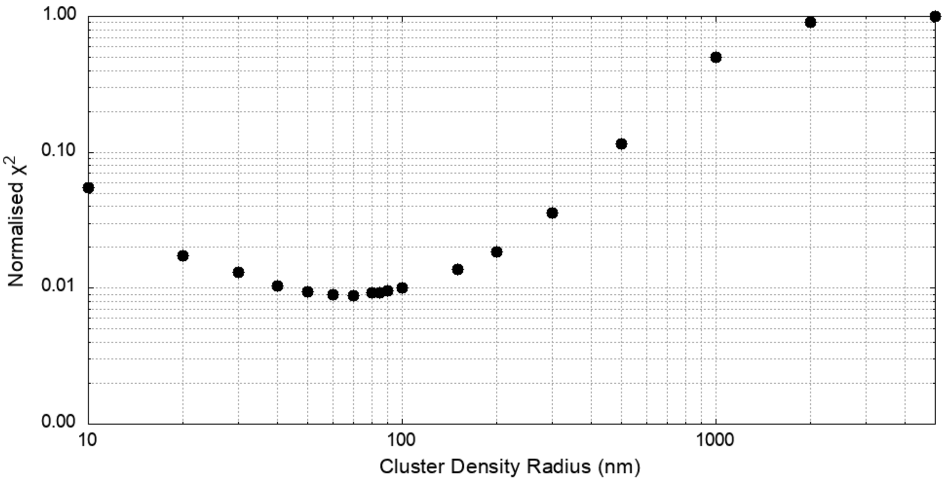

Figure S9. The goodness of fit between misrepair and cluster density calculated at a range of different radii. Showing the best fit for cluster densities calculated at 70 nm. However, the goodness of fit values are similar for radii below 100 nm.

6. Cluster Density and LET

The cluster density as a function of LET_t can be correlated with a second order polynomial. This is shown for protons, carbons, and alpha. Calculated with a radius of 70 nm:

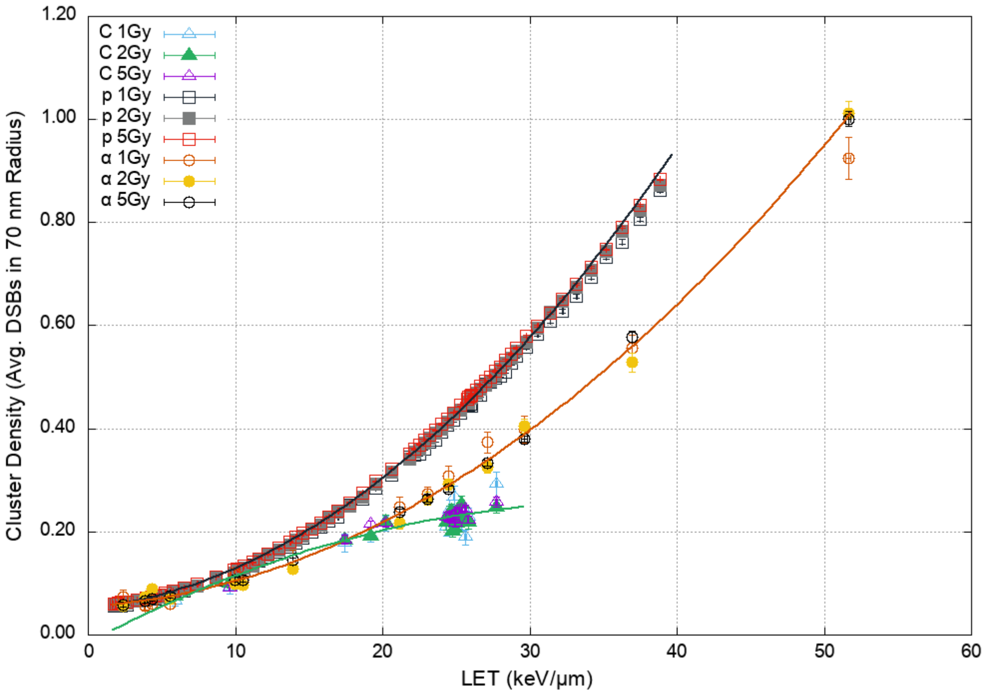

Figure S10. LET and cluster density for protons, alphas, and carbon ions. The cluster density can be approximated from a 2nd order polynomial, shown by solid lines.

7. Geant4-DNA and the Carbon ion discontinuity.

The results of this work have highlighted anomalous behaviour in the interactions of carbon-12^{6+} simulated through Geant4-DNA. For the default DNA physics list the only carbon interaction modelled is ionisation, handled by the "G4DNARuddIonisationExtendedModel". Within this model the kinetic energy transferred from the primary carbon ion to the liberated electron is calculated either relativistically or classically. This change in calculation method becomes apparent when calculating the primary carbon LET_t at a high primary energy resolution. However, it is possible to force Geant4-DNA to always use the relativistic calculation, which should still have validity at non-relativistic energies.

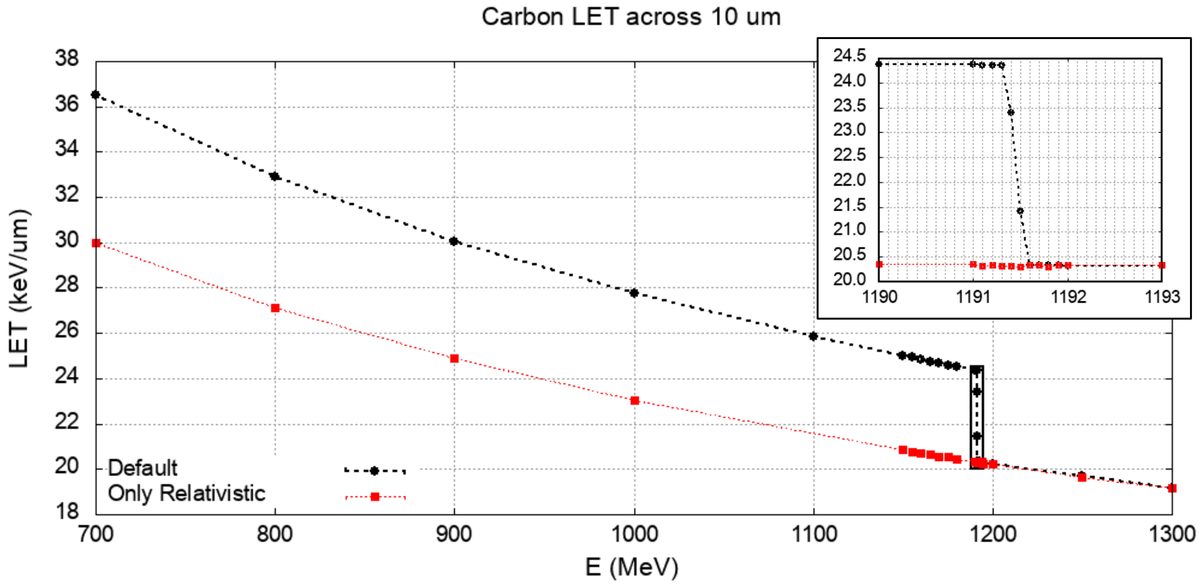

Figure S11. The carbon ion LET as a function of primary energy. Showing a discontinuity between 1191.3 MeV and 1191.6 MeV. The discontinuity is attributed to a change in calculation method, where Geant4 switches between classical and relativistic calculations.

Here we see the change in behaviour occurring between carbon ions at 1191.3 MeV and 1191.6 MeV. Forcing relativistic calculation removes the discontinuity. This has implications for any study using Geant4-DNA to simulate carbon ions at high LET_t, or approaching their Bragg peak. The energy transfer currently proposed by Geant4-DNA may be considerably lower than in reality.

8. Simulation results compared to experimental 53BP1 foci at 24 hours.

The amount of 53BP1 foci 24 hours post proton irradiation is extracted from Chaudhary *et al.*[61] and replotted alongside our prediction for residual DSBs at 24 hours. All points from "This Work" and for "Chaudhary *et al.* (2016)" are for 1 Gy, except the lowest LET in "Chaudhary *et al.* (2016)". The error bars for both data sets show the standard error in the mean.

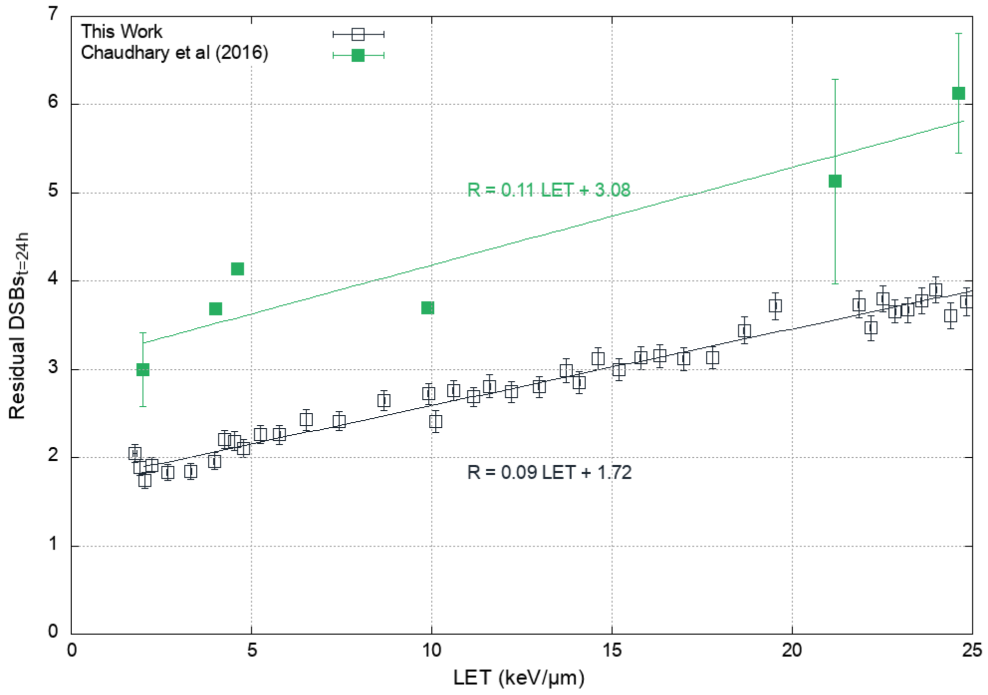

Figure S12. Experimental values of residual DSBs from Chaudhary *et al* compared to the predicted yield of residual DSBs following 24 hours of repair.

9. Incorrect Rejoining Time.

Our model predicts that misrepair occurs rapidly. This is shown for our model by scoring the times at which misrepair occurs for the case of various energies of 1 Gy protons. The majority of misrepair occurs before 95 seconds and there is no increase in the frequency of misrepair events after this time.

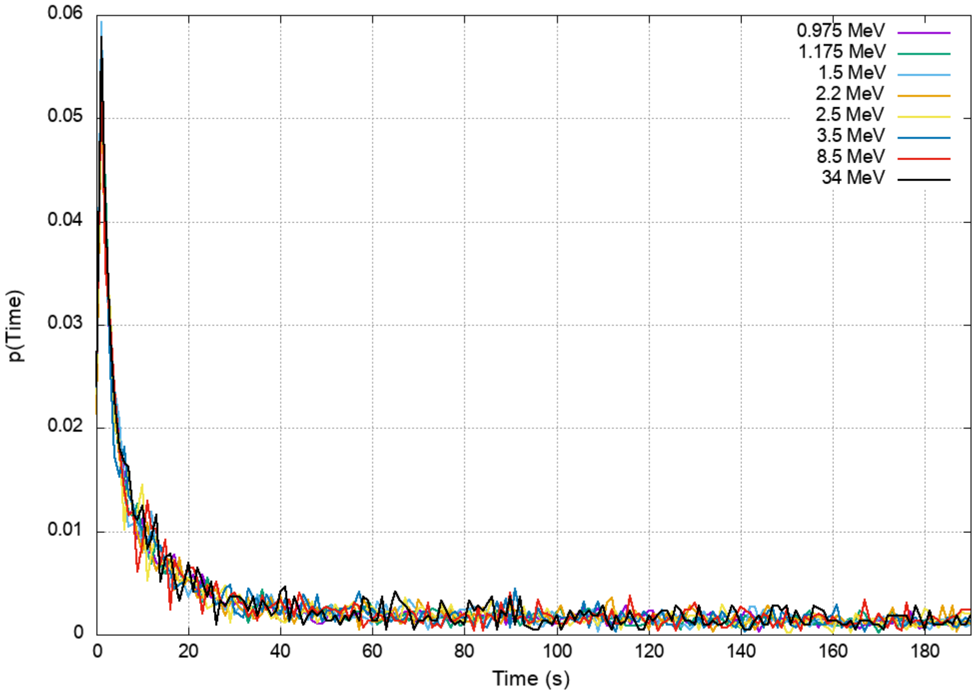

Figure S13. The time that misrepair occurs for DSBs created by a range of 1 Gy protons. Showing that repair of DSBs occurs rapidly, with the majority occurring before 95 seconds.
